# Cefcapene pivoxil-induced hypocarnitinemic hypoglycemia in elderly man with subclinical ACTH deficiency: a case report

**DOI:** 10.1186/s12902-023-01314-5

**Published:** 2023-03-06

**Authors:** Yoshihiro Takahashi, Masanori Murayama, Kaoru Noda, Kengo Yamakawa, Yuya Koide, Rie Yamada, Makoto Hayashi, Keigo Yasuda

**Affiliations:** 1 Department of Internal Medicine, Matsunami General Hospital, Dendai 185-1, Kasamatsu-cho, Hashima-gun, Gifu, 501-6062 Japan; 2 Department of Diabetes, Endocrinology and Metabolism and Department of Rheumatology and Clinical Immunology, Gifu University Graduate School of Medicine, 1-1 Yanagido, Gifu, 501-1194 Japan

**Keywords:** Hypoglycemia, Hypocarnitinemia, Hypopituitarism, Elderly, Frailty

## Abstract

**Background:**

Drug-induced hypocarnitinemia has been noted as a cause of hypoglycemia in children. However, adult cases are extremely rare and pre-existing conditions (including endocrine disorders and frailty) have been suggested to be involved. Hypoglycemia due to drug-induced hypocarnitinemia is quite rare, and there were few reports of pivoxil-containing cephalosporin (PCC)-induced hypocarnitinemia in adults.

**Case presentation:**

We present a case of an 87-year-old man with malnutrition, and frailty. He developed severe hypoglycemia with unconsciousness after taking cefcapene pivoxil hydrochloride, one of PCC, and hypocarnitinemia was diagnosed. Despite levocarnitine administration, asymptomatic mild hypoglycemia had persisted. Subsequent investigation revealed subclinical ACTH deficiency due to empty sella, which played a key role to maintain mild hypoglycemia as underlying disorder, and PCC-induced hypocarnitinemia triggered severe hypoglycemia. The patient responded to hydrocortisone therapy.

**Conclusions:**

We need to be aware of the facts that PCC can induce severe hypocarnitinemic hypoglycemia in elderly adults associated with frailty, malnutrition, and subclinical ACTH syndrome.

## Background

Hypoglycemia is considered to be uncommon in nondiabetic patients, occurring in < 1% of all hospital admissions [1], and the underlying causes are infection, liver disease, malignancies, chronic kidney disease, and drug, in order of incidence [2]. On the other hand, the causes of hypoglycemia in older patients appear to be different from those in younger patients, including malnutrition, malignancies, renal failure, and sepsis as the most common causes [1, 3], but endocrinopathy is quite unusual [1, 4].

Carnitine is an essential nutrient involved in fat metabolism, transporting the activated long chain fatty acids from the cytosol into the mitochondria, making them available for mitochondrial β-oxidation [5]. Hypocarnitinemia generally presents with hypoglycemia, loss of consciousness, and muscle weakness. Valproate [6] and pivoxil-containing cephalosporin (PCC) antibiotics [7] have been known to result in secondary hypocarnitinemia. Most reported cases of PCC-induced hypocarnitinemic hypoglycemia were reported in infants and children [7], and adult cases are extremely rare, and only two cases were reported [8, 9].

We report an elderly man with severe non-ketonic PCC-induced hypoglycemia associated with hypocarnitinemia. In this case, asymptomatic mild hypoglycemia had persisted even after levocarnitine administration, and subclinical adrenocorticotropic hormone (ACTH) deficiency [10] by empty sella was revealed. These findings showed a new pathophysiology of hypoglycemia that two independent pathological conditions linked to cause severe hypoglycemia. One is preceding subclinical ACTH deficiency for background mild asymptomatic hypoglycemia and the other is PCC-induced hypocarnitinemia for resulting in severe hypoglycemia in adult with malnutrition and frailty.

## Case presentation

An 87-year-old Japanese man was admitted to our hospital because of loss of consciousness. He had hypertension, atrial fibrillation, and angina. His regular medications did not include hypoglycemic drugs. At 18 months before admission, he was admitted to a nursing home due to cognitive impairment and gradually progression to frailty. One week before admission, he suffered from urinary tract infection with fever, and took 300 mg of cefcapene pivoxil hydrochloride, one of PCC, three times a day for 7 days. The next morning after the last dose of cefcapene pivoxil hydrochloride, he was found to be unconsciousness, and was referred to the emergency department, and hospitalized.

On arrival, the Glasgow coma scale was 10, and his vital signs and oxygen saturation were normal. He was lean (body weight, height and body mass index, 45.2 kg, 159 cm and 17.9 kg/m^2^, respectively), and his limbs were emaciated (the mid-upper arm and the thigh circumferences [right/left], 22.0/21.1 cm and 32.8/32.8 cm). Rest of physical examination findings were unremarkable, including normal body hairs. Blood glucose was 31 mg/dL, and urine ketone was negative. Intravenous administration of glucose immediately relieved his symptoms. After admission, all medications were discontinued.

His laboratory data showed hypoalbuminemia, hypocholesterolemia, and anemia (Table 1). Hemoglobin A1c (HbA1c) was 4.7%. Later, blood samples obtained at admission revealed hypocarnitinemia; free carnitine (FC):17.0 μmol/L, acylcarnitine (AC); 16.5 μmol/L, (normal reference ranges; FC; 36–74 μmol/L, and AC; 6.0–23.0 μmol/L, respectively). AC/FC ratio was 0.971. Hypocarnitinemia is diagnosed when FC level is < 20 μmol/L and AC/FC ratio of > 0.4 [9, 11]. Hypocarnitinemic hypoglycemia was diagnosed, and levocarnitine (2250 mg/day) was administered. However, low blood glucose levels (50–70 mg/dL) persisted without any symptoms. Plasma insulin level was undetectable, and C-peptide was low (Table 2). He was transferred to our department on the 12th day of hospitalization.

**Table 1:** Laboratory data on admission

Peripheral blood			AST	24	U/L
WBC	2900	/mm^3^	ALT	6	U/L
Neut	72.0	%	ɤ-GTP	9	U/L
Eosino	2.0	%	Total-chol	2.07	mmol/L
Baso	1.0	%	HDL-chol	0.67	mmol/L
Mono	10.0	%	LDL-chol	1.34	mmol/L
Lympho	15.0	%	Triglyceride	0.47	mmol/L
RBC	2.34 × 10^6^	/mm^3^	CRP	9.12 × 10^4^	ug/L
Hb	74	g/L	Glucose^a1^	13.1	mmol/L
Hct	0.211	/L	HbA1c	4.7	%
Plt	107 × 10^3^	/mm^3^	NH_3_	29.4	μmol/L
Blood chemistry			Urinalysis		
Sodium	135	mmol/L	pH	6.5	
Potassium	1.8	mmol/L	Glucose	negative	
Chloride	88	mmol/L	Ketone body	negative	
Calcium	2.18	mmol/L	Occult blood	negative	
Phosphorus	0.71	mmol/L	Specific gravity	1.008	
Magnesium	0.58	mmol/L	Blood gas analysis (ambulatory air)		
TP	64	g/L	pH	7.414	
Alb	24	g/L	PaCO_2_	58.4	mmHg
BUN	4.46	mmol/L	PaO_2_	28.6	mmHg
Cre	65.4	mmol/L	HCO_3_^−^	36.6	mmol/L
UA	374.7	umol/L	Lactate	1.9	mmol/L
Amy	50	U/L	Glucose	1.72	mmol/L
T-Bil	15.4	umol/L			

*WBC* White blood cells, *Neut* Neutrophil, *Eosino* Eosinophil, *Baso* Basophil, *Mono* Monocyte, *Lympho* Lymphocyte, *RBC* Red blood cells, *Hb* Hemoglobin, *Hct* Hematocrit, *Plt* Platelet, *TP* Total protein, *Alb* Albumin, *BUN* Blood urea nitrogen, *Cre* Creatinine, *UA* Uric acid, *Amy* Amylase, *T-Bil* Total bilirubin, *AST* Aspartate aminotransferase, *ALT* Alanine aminotransferase, *γ-GTP* γ-glutamyl transpeptidase, *CRP* C-reactive protein

^a^1: After correction

**Table 2:** Additional data (under fasting)

Glucose	3.55	mmol/L	Total carnitine	33.5	μmol/L
Insulin	< 1.0	pmol/L	Free carnitine	17.0	μmol/L
C-peptide	0.142	nmol/L	Acyl carnitine	16.5	μmol/L
Insulin antibody	< 0.4	U/mL			
**Endocrinological evaluations**					
ACTH (4–22)	4.38	pmol/L	Prolactin (< 20)	57.7	μg/L
Cortisol (110–520)	147	nmol/L	Vasopressin (<=4)	0.4	pg/mL
TSH (2–11)	1.186	mU/L	Plasma renin activity	0.31	ng/(L・S)
Free T4 (0.107–0.228)	0.093	pmol/L	Aldosterone	157	pmol/L
GH (<=5)	0.70	μg/L	Adrenaline (170–520)	<=5	pmol/L
IGF-1 (48–177)	<=4	ng/mL	Noradrenaline (1.27–2.81)	0.97	nmol/L
LH (3–25)	<=0.10	IU/L	Dopamine (<=79.55)	<=5	pmol/L
FSH (1–10)	0.07	IU/L	DHEA-S (0.35–7.16)	0.081	μmol/L

*ACTH* Adrenocorticotropic Hormone, *DHEA-S* dehydroepiandrosterone-sulfate, *FSH* follicle stimulating hormone, *IGF-1* insulin-like growth factor-1, *LH* luteinizing hormone, *TSH* thyroid stimulating hormone. The reference range is shown in parentheses

**Fig. 1:**
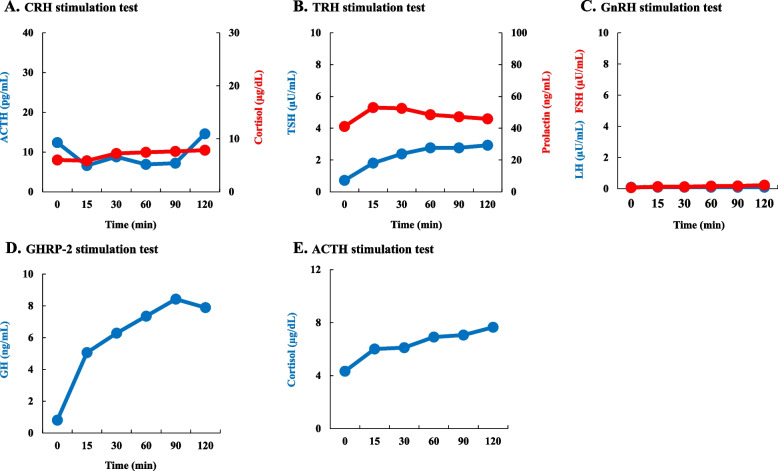
Results of endocrinological examinations. Various endocrinological stimulation tests suggest the presence of hypopituitarism. **A**. CRH stimulation test. **B**. TRH stimulation test. **C**. GnRH stimulation test. **D**. GHRP-2 stimulation test. **E**. ACTH stimulation test

**Fig. 2:**
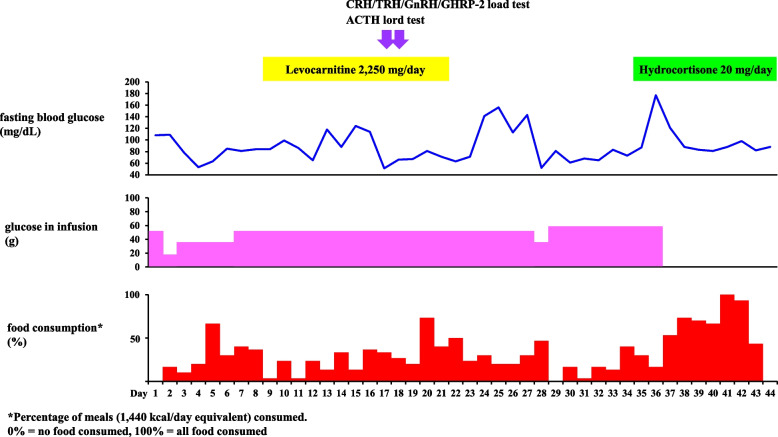
Changes of blood glucose, glucose in infusion and food consumption with treatment. After diagnosis of hypocarnitinemia, levocarnitine was started, but mild hypoglycemia persisted, requiring intravenous glucose supplementation After starting hydrocortisone, the intravenous infusion could be discontinued, and food consumption stabilized

The pituitary and adrenal hormones were evaluated (Table 2). Basal levels of plasma cortisol and ACTH obtained at 8 am were within normal limits, and growth hormone (GH), insulin-like growth factor-1 (IGF-1), luteinizing hormone (LH) and follicle stimulating hormone (FSH) values were low or undetectable. Plasma thyroid stimulating hormone (TSH) and free thyroxine were slightly lower than normal limits (Table 2). In corticotropin-releasing hormone (CRH), thyrotropin-releasing hormone (TRH), gonadotropin-releasing hormone (GnRH), and GH releasing-peptide-2 (GHRP-2) stimulation tests, peak plasma ACTH, TSH, LH, FSH, and GH responses except prolactin were low (Fig. 1). Peak value of plasma cortisol to intravenous 250 μg ACTH was low (Fig. 1). Head magnetic resonance imaging revealed an empty sella. Subclinical ACTH deficiency with hypopituitarism was diagnosed due to empty sella. After diagnosis, hydrocortisone 20 mg/day was started. Immediately, his food intake trended upward and his hypoglycemia resolved (Fig. 2). The patient was discharged to a nursing home on day 44 of hospitalization.

## Discussion and conclusions

We reported an 87-year-old Japanese male with severe non-ketonic hypoglycemia with loss of consciousness after 7 days of cefcapene pivoxil hydrochloride administration, and hypocarnitinemia was confirmed. However, mild hypoglycemia had persisted despite daily carnitine administration, suggesting the association with other cause(s). Further hormonal studies revealed the presence of subclinical ACTH deficiency [10] due to empty sella as background disease, and complete recovery from hypoglycemia by hydrocortisone supplementation strongly supported these findings. This case demonstrated a new etiology of hypoglycemia through two independent causes, that is, subclinical ACTH deficiency with empty sella, and hypocarnitinemia originated by PCC administration. The former played a key role for maintenance of mild hypoglycemia, and the latter for exacerbation of mild hypoglycemia, resulting in severe hypoglycemia. This is probably the first clinically proven case of severe hypoglycemia with etiology by two independent causes.

Hypocarnitinemia-induced hypoglycemia is primarily caused by 1) decreased activity of pyruvate carboxylase in skeletal muscle mitochondria 2) impaired fatty acid oxidation due to impaired long-chain fatty acid transport into the mitochondrial matrix, and 3) reduction of free CoA. Carnitine specifically forms acetylcarnitine from acetyl-CoA, an essential substance for the action of pyruvate carboxylase in skeletal mitochondria. Therefore, the decrease in pyruvate carboxylase activity with hypocarnitinemia leads to impaired glycogenesis [12]. The mechanism of PCC-induced hypocarnitinemia is caused by increased urinary excretion of carnitine. The absorbed PCC is rapidly hydrolyzed in the small intestine to pivalate and an active antibiotic. Pivalate binds to free carnitine in the blood to become pivaloyl carnitine, which is excreted in the urine [13].

Symptoms of hypocarnitinemia are hypoglycemia, loss of consciousness, muscle weakness, cramp, and encephalopathy. The most common drug-induced hypocarnitinemia is caused by valproate administration [6], and PCC-induced hypocarnitinemia is observed in children, especially in infants [7]. Infants have only one-quarter of the adult ɤ-butyrobetaine dioxygenase activity required for carnitine biosynthesis and are most likely to develop carnitine deficiency. For this reason, infant carnitine levels are primarily dependent on oral intake. In addition, most carnitine is stored in the skeletal muscle, which accelerates the development of hypocarnitinemia in children with low skeletal muscle mass [13]. Therefore, hypoglycemia due to PCC-induced hypocarnitinemia is very rare in adults [8, 9].

In the present case, with exception of prolactin, basal levels of pituitary hormones other than ACTH and their responses to stimulation substances were low (Table 2). On the other hand, basal levels of plasma cortisol and ACTH were low but within normal limits, confirmed subclinical ACTH deficiency with hypopituitarism [10]. There were no signs and symptoms of hypoadrenocorticism including low blood pressure, decrease in body hair and eosinophilia (Table 1). The clinical course of the patient is consistent with the facts that hypopituitarism due to empty sella may develop insidiously, and ACTH deficiency eventually develops later in the course of pituitary failure [14]. In the present case, subclinical ACTH deficiency was identified as an underlying cause of preceding mild hypoglycemia. In general, some hypoglycemic episodes are mild and/or asymptomatic and may not be reported. Furthermore, aging affects the counter-regulation of glucose levels, and the glucose counter-regulation to hypoglycemia by both glucagon and epinephrine is impaired even in healthy elderly people [15]. In this case, it is likely that asymptomatic mild hypoglycemia was usually occurring. In fact, HbA1c (4.7%) derived average glucose (ADAG) was 88.2 mg/dl, which was slightly lower than normal range [16]. PCC-induced hypocarnitinemic hypoglycemia is a short-term event due to PCC administration, and it is not reflected in HbA1c. The observed low HbA1c and ADAG are an important clinical marker indicating the presence of some underlying disorder. In the two previously reported adult cases of PCC-induced hypocarnitinemic hypoglycemia, HbA1c and ADAG were 6.1% and 128.4 mg/dL [8], and 5.4% and 108.3 mg/dL [9], respectively. These data indicate that the mechanism of severe hypoglycemia in the present case is clearly different from that in the previously reported adult cases [8, 9]. HbA1c and ADAG should be always measured and calculated in non-diabetic hypoglycemia.

Adults with mature liver and skeletal muscles rarely develop hypocarnitinemia and subsequent hypoglycemia. However, blood carnitine level is also known to decrease with age [17]. In addition, our case had malnutrition and frailty with hypoalbuminemia, and hypocholesterolemia (Table 1). Normal daily L-carnitine requirement is about 15 mg, 25 and 75%, which comes from endogenous biosynthesis and exogenous sources, respectively. The main source of L-carnitine is red meat, especially lamb and beef [18]. Three adult cases of PCC-induced hypoglycemia including this case are all Japanese over 80 years of age, and they had malnutrition with frailty. The average consumption of beef in Japan is only 7.0 kg/year/person, about a quarter of USA [19]. Furthermore, it is expected that the intake of beef may be even lower in residents of nursing home and long-term care facilities. PCC-induced hypocarnitinemic hypoglycemia in adults may be a disorder peculiar to the elderly adults in countries with low beef intake. However, in a recent epidemiologic surveillance study of the FDA adverse event reporting system, one of PCC antibiotics, cefditoren, was the most associated antibiotic with hypoglycemia among various antibiotics used in USA, and the reported odds ratio for hypoglycemia was very high, 14.08 [20]. There may be an unreported adult case of cefditoren-induced hypocarnitinemic hypoglycemia.

Serum carnitine levels are reduced in malnourished adults and patients with hypopituitarism [21, 22]. It seems to be likely that various morbidity such as malnutrition, frailty and subclinical ACTH deficiency give an impact on the development of hypocarnitinemia due to PCC in adults. Thus, PCC might be regarded as a potentially inappropriate medication in elderly adults with these multi-morbidities. However, coadministration of levocarnitine with PCC may be one of the solutions in such patients. Accumulation of additional cases could provide further insights into the pathophysiological roles of malnutrition and frailty in elderly adults as potential risk factors for PCC-induced hypocarnitinemic hypoglycemia.

This case has several limitations. First, blood ketones should measure to prove hypoketonemia. However, the blood sample (on arrival at the hospital) already lost and could not be measured. On the other hand, the negative of urinary ketone body is an unlikely finding in typical hypoglycemia, and it suggests the presence of hypocarnitinemia. In addition, measurement of urinary carnitine was necessary to confirm renal excretion of carnitine. Unfortunately, we cannot measure that on a commercial basis. It is hoped that this fact will spread and contribute to the development of measurement technology.

We present the case of 87-year-old non-diabetic man with severe hypoglycemia which was developed through two independent causes, subclinical ACTH deficiency due to empty sella as a background disorder for maintenance of mild hypoglycemia, and PCC-induced hypocarnitinemia for exacerbation of mild hypoglycemia. This case shows that PCC can induce hypocarnitinemic hypoglycemia even in adult patients, when they have some morbidity such as aging, malnutrition, frailty etc. In such cases, coadministration of PCC with levocarnitine may be one of the solutions for avoidance of hypoglycemia.

## Data Availability

Clinical data from the corresponding author will be available upon request.

## Abbreviations

AC: Acylcarnitine
ACTH: Adrenocorticotropic Hormone
ADAG: A1c derived average glucose
FC: Free carnitine
FSH: Follicle stimulating hormone
HbA1c: hemoglobin A1c
IGF-1: Insulin-like growth factor-1
LH: Luteinizing hormone
PCC: Pivoxil-containing cephalosporin
TSH: Thyroid stimulating hormone
